# *Cis*-Regulatory Logic Produces Gene-Expression Noise Describing Phenotypic Heterogeneity in Bacteria

**DOI:** 10.3389/fgene.2021.698910

**Published:** 2021-09-28

**Authors:** Debajyoti Chowdhury, Chao Wang, Aiping Lu, Hailong Zhu

**Affiliations:** ^1^ HKBU Institute for Research and Continuing Education, Shenzhen, China; ^2^ Computational Medicine Lab, Hong Kong Baptist University, Hong Kong, China; ^3^ Institute of Integrated Bioinformedicine and Translational Sciences, School of Chinese Medicine, Hong Kong Baptist University, Hong Kong, China

**Keywords:** gene expression noise, combinatorial regulation, promoter architecture, logic gates, phenotypic heterogeneity

## Abstract

Gene transcriptional process is random. It occurs in bursts and follows single-molecular kinetics. Intermittent bursts are measured based on their frequency and size. They influence temporal fluctuations in the abundance of total mRNA and proteins by generating distinct transcriptional variations referred to as “noise”. Noisy expression induces uncertainty because the association between transcriptional variation and the extent of gene expression fluctuation is ambiguous. The promoter architecture and remote interference of different *cis*-regulatory elements are the crucial determinants of noise, which is reflected in phenotypic heterogeneity. An alternative perspective considers that cellular parameters dictating genome-wide transcriptional kinetics follow a universal pattern. Research on noise and systematic perturbations of promoter sequences reinforces that both gene-specific and genome-wide regulation occur across species ranging from bacteria and yeast to animal cells. Thus, deciphering gene-expression noise is essential across different genomics applications. Amidst the mounting conflict, it is imperative to reconsider the scope, progression, and rational construction of diversified viewpoints underlying the origin of the noise. Here, we have established an indication connecting noise, gene expression variations, and bacterial phenotypic variability. This review will enhance the understanding of gene-expression noise in various scientific contexts and applications.

## Introduction

Genetically identical cells often behave differently despite sharing consistent growth conditions (Sanchez et al., 2013; Jones et al., 2014; Engl, 2019). Bacteria adapt rapidly to environmental pressures to attain competitive survival advantages in detrimental conditions (Roberfroid et al., 2016; Engl, 2019). Such competency in modulating phenotypic plasticity is the major driver influencing such variability (Roberfroid et al., 2016). Transcriptional noise usually drives heterogeneous gene expressions, and thus determines the phenotypic fate of each cell (Coskun et al., 2016; Engl, 2019; Iida et al., 2019) and empowers some bacteria to become more adaptive (Imdahl et al., 2020; Sun et al., 2020).

**FIGURE 1:**
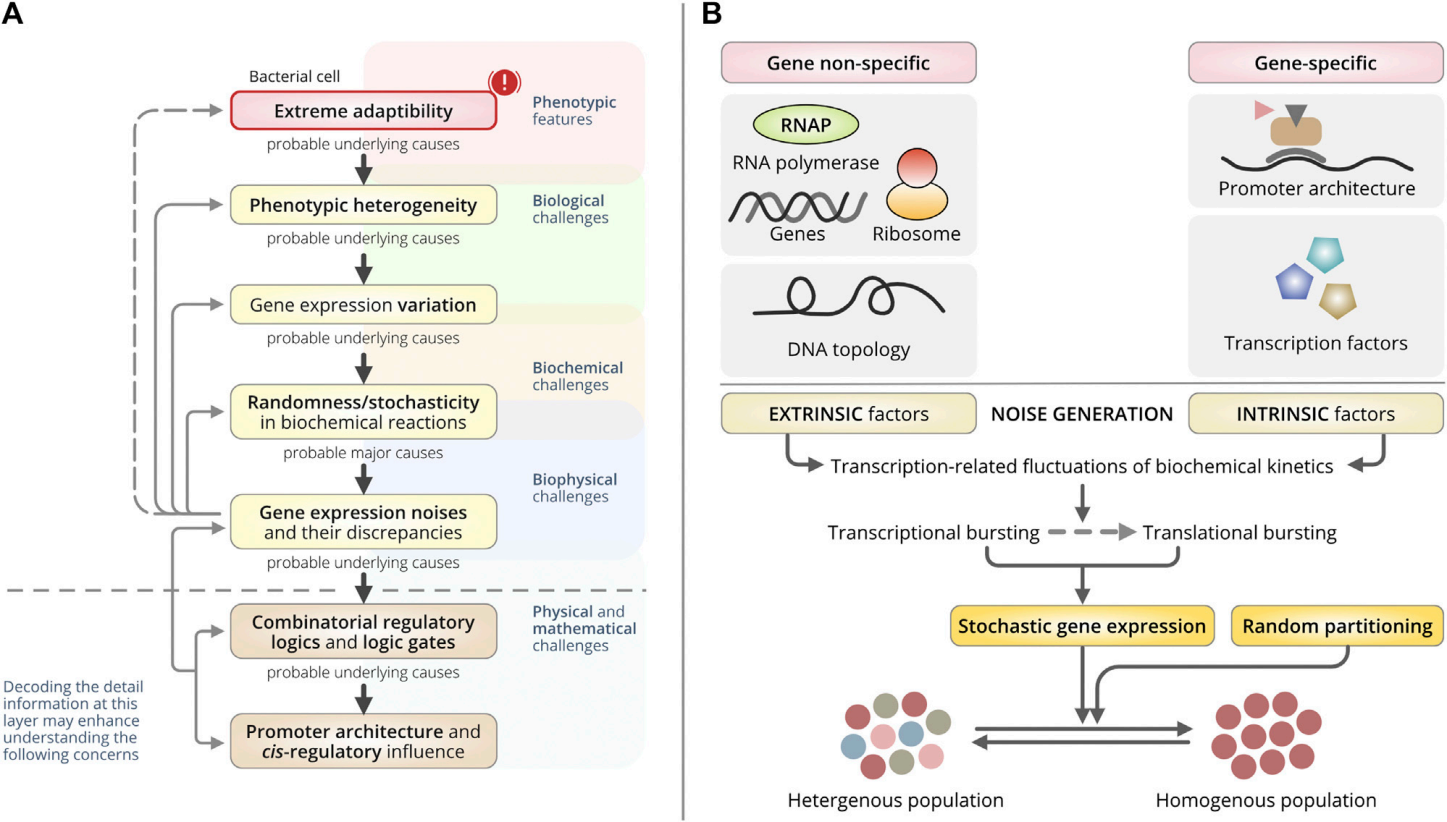
Overall concept and origin of gene expression noise. **(A)** A schematic dogma of the generation of bacterial phenotypic heterogeneity. This schematic represents the central direction and the overall realizations by the authors discussed in this review paper. Biological challenges are greatly masked by the multiplexed layers backed by biological origin, biophysical origin, and deep-rooted physical and mathematical origin. Similarly, bacterial heterogeneity and random yet extreme adaptability in a wide range of detrimental circumstances have been a great challenge. These challenges are merely not to be addressed by biological knowledge only. The underlying mechanisms, quantitative foundations, structural and functional aspects including their interdependencies to each other are inevitably pivotal to trace back those intricated challenges. In this review, the authors have identified the relational model of the phenomenon from the very molecular level to the phenotypes. (Top to bottom direction): A few challenges with bacterial extreme adaptability and prompt phenotypic switching can be explained with the basis of phenotypic heterogeneity. To reveal the basis of phenotypic heterogeneity, the underlying biological challenges, that is the variations in gene-expressions are essential to understand. And, the differential gene-expression noise, intrinsic and extrinsic transcriptional noise, and their discrepancies may support the explanation of the wide extent of gene-expression variation. It relies mostly on the biophysical basis of gene expression. Subsequently, a question remains important that what drives or controls those noises? The most probable hints have been established with the arising scientific supports enriching the underlying causes as the different regulatory logics and different combinations of the logic gates being constructed by the different regulators. Finally, the promoter architecture and the successive interaction of different *trans-*regulatory elements have been identified to influence the combinatorial selection of different logic gates that triggers the variable gene-expression noise (bottom to top), thus the variation in gene-expressions and causing bacterial phenotypic heterogeneity. **(B)** Schematic of the origin of gene-expression noise that dictates the cell-to-cell variation in the clonal population of bacteria. Bacterial cell-to-cell variability in the clonal population used to be mediated through stochastic gene expressions or fluctuations in random partitioning during cell division. Stochastic gene expression is majorly influenced by gene-independent extrinsic factors and gene-specific or gene-dependent intrinsic factors through gene-expression related fluctuations in biochemical reaction kinetics. It gets manifested in both transcriptional and translational bursting.

Randomness arising from biochemical reactions during transcription is one of the major drivers underpinning gene-expression noise (Sanchez et al., 2013). It is directly influenced by the *cis*-regulatory elements (CREs). CREs are regulatory DNA sequences that include enhancers and promoters that regulate gene expression on the same chromosome (Park and Wang, 2018). Noise can also be generated during translation when individual mRNAs within a cell fluctuate randomly (Bar-Even et al., 2006; Silander et al., 2012; Espinar et al., 2017). However, these are not gene-specific and, thus, do not directly regulate CREs. In this review, we focused on the manifestations of transcriptional noise affecting gene expression and phenotypic variability (Figure 1A). An understanding of the interactions among CREs and their influence on transcription may help to elucidate the mechanisms by which bacteria produce diverse phenotypes (Imdahl et al., 2020).

Combinatorial gene regulation by multiple transcription factors (TFs) is highly utilized in cells in responding to environmental conditions, enables the organism to generate diverse expression patterns facilitated by a limited number of TFs. It can be characterized using the concept of logic gates (Yan et al., 2017), as this illustrates the diverse combinations of TFs, CREs, and other regulators in the remote promoter region (Sepúlveda et al., 2016). The transcription initiation rate is a key determinant of transcriptional regulation (Lionnet and Wu, 2021). Diversified interaction patterns among those limited number of regulators against a single binding site introduce randomness in the system, and thus the higher level of noise (Yan et al., 2017). The physical architecture of the promoter region and the influence of CREs support the understanding of combinatorial associations, although the mechanism is poorly understood (Sun et al., 2020; Mejía-Almonte et al., 2020; Morrison et al., 2021).

We lack a complete insight into the transcriptional machinery embedded in deep cellular biophysics that is associated with different biological challenges (Sun et al., 2020; Mejía-Almonte et al., 2020). Most of our knowledge is derived from static biochemical experiments (Park and Wang, 2018; Imdahl et al., 2020), which usually fail to consider the versatility of transcriptional machinery and the stochastic interactions among regulators (Park and Wang, 2018; Imdahl et al., 2020). These approaches are limited by sharing interdisciplinary efforts to address such multimodal challenges (Figure 1A). So, it becomes imperative to decode the transcriptional mechanisms (Sanchez and Golding, 2013; Imdahl et al., 2020). Integrated frameworks with quantitative modeling are one of the proficient ways to expand experimental knowledge to develop novel testable hypotheses. Recent advancements in high-throughput sequencing, multi-omics technologies, and genomic data science have significantly augmented our knowledge. For example, bacterial single-cell RNA sequencing has enabled the study of CRE-driven gene-expression noise in an individual cell revealing the CRE-mediated interaction patterns (Hermsen et al., 2006; Sánchez and Kondev, 2008; Yan et al., 2017; Imdahl et al., 2020). Such advancements, combined with an effective modeling approach, can decode the underlying forces causing gene expression variations producing phenotypic heterogeneity (Figure 1A).

## Transcriptional Regulatory Switches in Bacteria

Bacterial transcriptional regulation is primarily governed by operons (Müller et al., 1996; Bintu et al., 2005a; Kaern et al., 2005; Sánchez and Kondev, 2008; Kokubo, 2013; Sanchez et al., 2013; Rocabert et al., 2020). It consists of four components: a regulatory gene, operator(s), promoter, and structural genes (Conway et al., 2014). The discovery of the *lac*-operon model aided to decode complex coordination in bacteria (Esmaeili et al., 2015). In bacteria, gene regulation primarily facilitates adjustment and adaptation to nutritional changes aiding their optimized growth (Blake et al., 2006; Cheng et al., 2017). There are no genes continuously active. Local growth conditions and metabolism requirements induce gene transcriptions (Blake et al., 2006; So et al., 2011) through numerous proteins (complex or individual) that influence other regulatory proteins. The transcriptome contains signaling information that facilitates the recognition of the genes to be activated (Taniguchi et al., 2010; Sanchez et al., 2013; Wang et al., 2015). Therefore, a thorough study of the bacterial transcriptome may elucidate the core mechanisms supporting those heterogeneous instructions.

### *Cis*-Regulatory Controls in Bacterial Transcription

CREs act proximally at their target genes and serve as anchoring sites for numerous proteins that influence their adjacent genes (Cheng et al., 2017). CRE mutations occurring at the structural genes of the *lac*-operon restrict the *lac*-repressor from anchoring to the operator, and CRE mutations in the *lac-*promoter induce structural changes in RNA polymerase (RNAP) binding sites that inhibit transcriptions (Choudhary and Narang, 2019). Also, *trans*-acting elements (TREs) sometimes control gene expression on distal DNA molecules, translating diffusible proteins, and occasionally RNAs (Park and Wang, 2018). They influence gene expression in *trans*.

### Combinatorial Regulatory Logic in Bacterial Transcription Regulation

**FIGURE 2:**
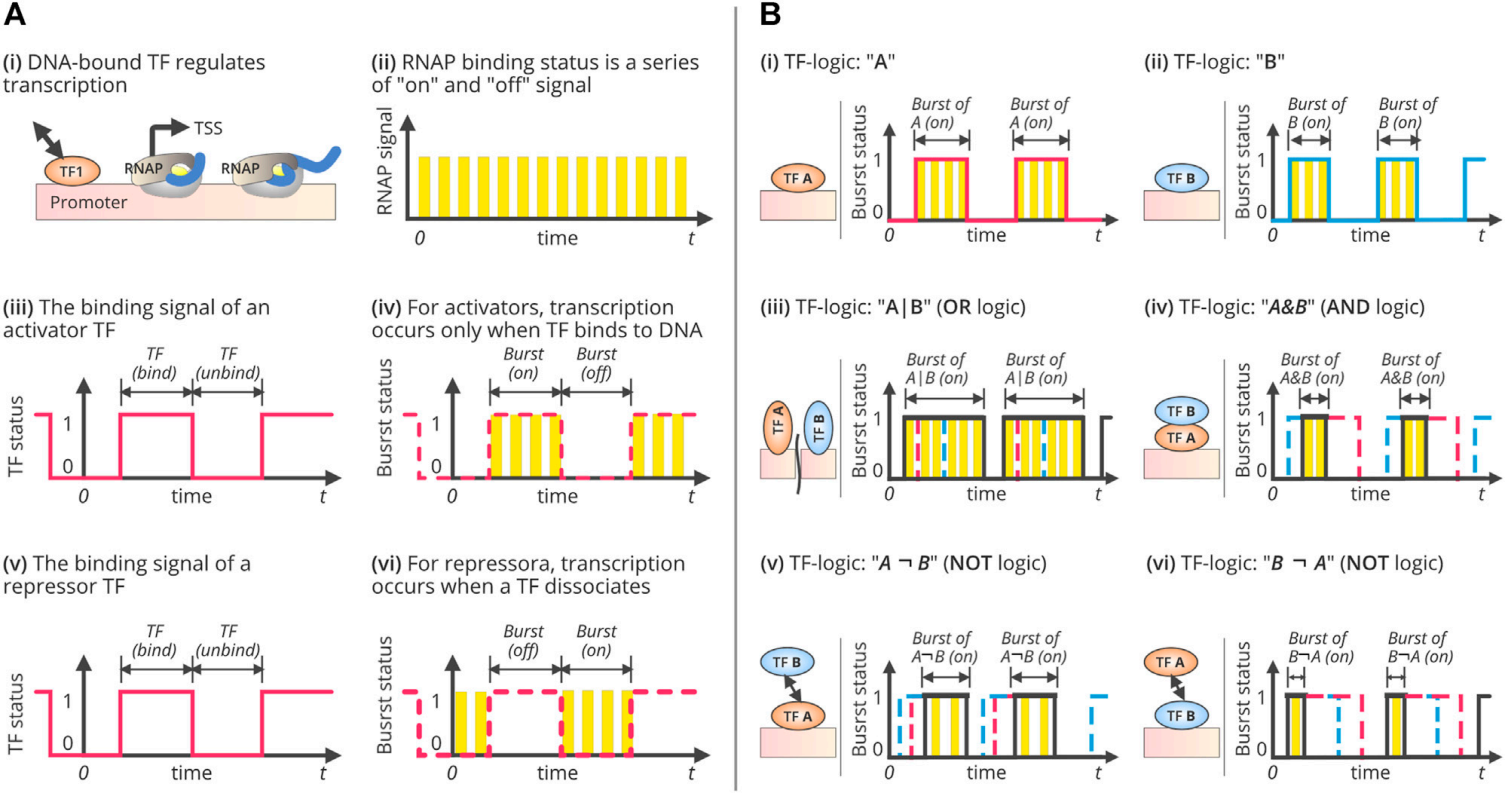
TF-driven gene regulation, transcriptional bursting, and combinatorial regulatory TF-logics. **(A)** Single-TF regulation and transcriptional bursting. It illustrates the process of gene transcription under single-TF regulation. During transcription, RNAP binding (i) can be viewed as a series of on/off signals, as shown in (ii). Each “on” status is corresponding to a transcript initiation. Therefore, the frequency of the “on” events are equivalent to the rate of transcript initiation, as well as the mRNA synthesis rate if every synthesized pre-RNA is converted into a mature mRNA. Similarly, the TF binding status can also be viewed as a digital signal with a series of “bind” and “unbind” events. The TF “bind” state lasts from the moment of TF binding to the time of TF-DNA dissociation. The duration of TF binding depends on the relative affinity of its competitors, such as other DNA binding molecules. The “unbind” state, however, refers to the status that the promoter is not bound by the TF. The duration of the “unbind” state is the time that TF spends searching for the binding site after TF-DNA dissociation. Since the transcription can only be initiated during TF binding (in case that the TF is an activator), or during the process of TF disassociation (in case that TF is a repressor), thus making the transcription occurs in bursts. (iii) and (iv) show the binding signal of an activator TF and the transcriptional bursts, while (v) and (vi) show the binding signal of a repressor TF and the bursts. **(B)** Three fundamental combinatorial logics of gene regulation and their bursting patterns. Three fundamental logics of combinatorial gene regulation and the patterns of transcriptional bursts. It illustrates the process of gene transcription under two-TFs regulation while forming three basic combinatorial logics, OR, AND, and NOT. In systems biology, the regulatory interactions between TFs are typically represented as logic, such as OR (denoted by “|”), AND (“&”), and NOT (“¬”). This figure shows several logics and the corresponding bursting patterns, (i) while the TF, “A” acts as an activator; (ii) while the TF, “B” acts as an activator; (iii) while the TF, “A” and “B” cooperate with OR logics by forming, A|B logic gates; (iv) while the TF, “A” and “B” cooperate with AND logics by forming, A&B logic gates; (v) while the TF, “A” and “B” interacts with NOT logics by forming, A¬B logic gates, implies that “A” should be present to activate the transcription but “B” should not be present; and, (vi) while the TF, “B” and “A” interacts with NOT logics by forming, B¬A logic gates, implies that “B” should be present to activate the transcription but “A” should not be present. Specifically, two TFs can form eleven logics, including, (1) no TF needed (“None”); (2) one single activator “A”; (3) one single activator “B”; (4) one single repressor “A*”; (5) one single repressor “B*”; (6) “A” or “B” can independently activate gene transcription, denoted as A|B; (7) “A” and “B” need work cooperatively to activate the gene, i.e. A&B; (8) “A” is an activator while “B*” is a repressor, i.e. A¬B*; (9). “B” is an activator and “A*” is a repressor, i.e., B¬A*; (10) “A” or “B” can independently repress gene transcription, i.e., A*|B*, and (11) “A” and “B” need work cooperatively to repress gene transcription, i.e. A*&B*.

Combinatorial associations of different regulators tend to form diverse logic gates which are essential for bacterial transcription (Kuhlman et al., 2007; Lin et al., 2015; Scholes et al., 2017; Reiter et al., 2017). Logic gates are designated as the overlying arrangements of cooperative binding sites with an additional regulatory layer that represents different interaction modules. Transcriptional signals are aggregated through the interplay between intramolecular cooperative interactions and intermodular competition (Hermsen et al., 2006). It facilitates the mapping of multiple inputs to one output (Silva-Rocha and de Lorenzo, 2008; Yan et al., 2017; Bordoy et al., 2019; Mejía-Almonte et al., 2020). This represents the programming of input signals, which typically varies as per the temporal concentrations of TFs, and the output signals used to signify the expression levels of the target genes (Yan et al., 2017). Eventually, they stipulate the operon status for different TF concentrations (Hermsen et al., 2006). Figure 2B illustrates the typical combinations of logic gates comprising two different TF concentrations. The rationale of these processes relies on the design and microarchitecture of the *cis*-regulatory domain wherein the arrays of binding sites often overlap and compete (Hermsen et al., 2006). This architecture is certainly complex. In this connection, a few predictive models attempted to understand the balance between intramodular and intermodular competition upon combining several physio-chemical parameters within the evolutionary algorithm (Hermsen et al., 2006; Sanchez et al., 2013). In *Versatility of Gene-Expression Noise*, we discuss how certain models aid researchers in this endeavour in greater detail.

## Bacterial Gene-Expression Noise and its Origin

Noise primarily relies on fluctuations in gene expression correlated with oscillations in DNA topology and promoter architecture (Engl, 2019) (Figure 1B). They characterize the origin of noise as either extrinsic or intrinsic (Elowitz et al., 2002). Extrinsic noise is gene-independent and non-specific to gene expression, while intrinsic noise is gene-specific. Usually, the noise is introduced as a consequence of the innate stochasticity that occurs during transcription and translation. But the spans of both fluctuations used to vary (Rosenfeld et al., 2005), and these variations in gene transcription dictate phenotypic heterogeneity in bacteria (Engl, 2019) (Figure 1B).

Noisy transcription occurs across the entire bacterial genome, as confirmed by protein abundance profiling of a single *E. coli* cell (Taniguchi et al., 2010; Tyagi, 2010). The extrinsic noise usually exhibits a higher abundance of proteins (>10) per cell, whereas the intrinsic noise is used to exhibit a lower abundance of proteins (<10) per cell (Taniguchi et al., 2010; Tyagi, 2010). And the promoters governing the expression of essential genes exhibit low levels of noise (Silander et al., 2012) implying the homogenous expression of essential genes (Silander et al., 2012). Wherein, the essential proteins are usually expressed in higher abundance, indicating that extrinsic noise is critical in bacteria.

## Gene Expression Variability in Clonal Populations

In prokaryotes, a single parent cell undergoes binary fission to generate a population of cells known as clonal populations (Viney and Reece, 2013). So, they are anticipated to possess identical genotypes, but this is not always the case. Genotypes within clonal populations used to differ significantly (Viney and Reece, 2013; Roberfroid et al., 2016). Transcriptional variations among them trigger cell-to-cell variations that produce phenotypic heterogeneity (Jones et al., 2014). Disparities in gene expression and regulation, such as noise (intrinsic and extrinsic) and bistability to specific responses to variations in the molecular environment, are two prominent mechanisms imparting variations in a clonal population termed phenotypic plasticity (Roberfroid et al., 2016). It is predominantly random, non-programmed, multivariable, and stochastic events (Viney and Reece, 2013; Roberfroid et al., 2016). It undergoes clonal evolution mediated by mutations and copy number variations and thus provides an added source of variability in gene expression (Smith et al., 2016). However, the mechanisms by which a few molecular regulators and CREs control such wide phenotypic variability remains unknown. Thus, research must consider the origin of these stochastic fluctuations and may untangle some of the complexity, allowing us to understand and manipulate the origin of divergent phenotypes.

## Versatility of Gene-Expression Noise

The versatility of gene-expression noise induces phenotypic variations. And the abundance of TF-binding sites (TFBS) significantly influences the noise (Sanchez et al., 2013). The accurate mechanism of action has not yet been elucidated. Transcriptional regulatory mechanisms and the abundance of biochemical variability may generate the magnitude of distinctive signature noise corresponding to heterogeneous gene expression (Sanchez et al., 2013). Certain controversies prevail around the relationship between signature noise and the noise-to-mean trend line (Ozbudak et al., 2002; Sanchez et al., 2013). A noise-to-mean trend line is followed by a large number of genes, which contradicts the generation of transcriptional regulation-driven signature noise. This contradicts the concept of noise, which is regulated in the promoter region or by the DNA sequence of the regulatory regions (Ozbudak et al., 2002; Sanchez et al., 2013).

Multiple layers of transcriptional regulation are deeply intertwined (Mejía-Almonte et al., 2020). Studies suggested a cohesive justification for this universal relationship that is dominant in bacteria and yeast (Balaji et al., 2006; To and Maheshri, 2010; Larson et al., 2011) and specify essential functions of the promoter architecture in regulating noise. Several promoter-driven noises have also drifted considerably from a universal relationship that endorses the predominant roles of the promoter architecture and promoter architecture-specific elements in determining noise (Sanchez et al., 2013).

### Promoter Architecture

Promoter architecture determines the degree of gene-expression noise (Jones et al., 2014; Einav and Phillips, 2019; Engl, 2019; Huminiecki and Horbańczuk, 2020). The promoter architecture was found to determine the promoter-specific noise in *E. coli* (Silander et al., 2012), which contradicts the hypothesis of extrinsic noise generation. Earlier evidence was not sufficient to discriminate the effects of promoter-specific influence and induction conditions on gene expression (Jones et al., 2014; Einav and Phillips, 2019; Engl, 2019; Huminiecki and Horbańczuk, 2020; Imdahl et al., 2020). A single-molecule study also highlighted the impact of promoter architecture on transcription variation (Jones et al., 2014). Transcriptional noise fluctuates *in vivo* upon altering promoter-specific parameters, such as the concentration and binding affinity of repressors (Jones et al., 2014). This demonstrated that modulation of physical parameters can modify promoter architecture and induce fluctuations in gene expression. The authors also demonstrated the capacity of mutations within the regulatory DNA to alter transcriptional noise to induce transcriptional variations (Jones et al., 2014). Another study showed that the concentration of λ the -bacteriophage repressor introduced variations in the lysogeny maintenance promoter, P_RM_-derived stochastic expression (Sepúlveda et al., 2016). These promoter-specific behaviors were investigated in *E. coli*. The TF concentrations and abundance of synthesized mRNAs were estimated. They were mathematically modeled to identify the stochastic features of regulated promoters. The promoters rapidly switched their configurations (Sepúlveda et al., 2016). These switches were reported to be more frequent than the lifetime of mRNA synthesis in the same cell. Therefore, noise is a highly adjustable feature that is dependent on evolutionary selection pressure. These studies clarified the limitations of the previously proposed universal model that describes the ubiquitous nature of transcriptional noise (Jones et al., 2014; Sepúlveda et al., 2016).

### Promoter Architecture and Gene-Expression Noise in Bacteria

Promoter architecture-specific elements are a promising approach for characterizing the transcriptional noise. Despite considerable research, the extensive roles of TFs in transcriptional dynamics remain unclear. Previous studies have highlighted the mechanisms of multiple TF-mediated gene regulation (Browning and Busby, 2004; Morrison et al., 2021) which consists of deterministic biochemical kinetics. Then, several mechanisms have been tested *in vitro* and *in vivo* to estimate the level of gene expression as a function of available TF concentrations (Dodd et al., 2004; Bintu et al., 2005b; Kuhlman et al., 2007; Garcia and Phillips, 2011; Garcia et al., 2012; Ahsendorf et al., 2014; Morrison et al., 2021). This strengthens the construction of additional mathematical models accounting for all feasible states a promoter can adopt. The maximum number of such states used to be directly proportional to the occupancies of TFs and other regulators, including RNAPs at the promoter site (Bintu et al., 2005a; Stanton et al., 2014). The occupancies of these factors critically determine the typical fates of transcriptional activities and their regulation. Altogether, they used to be reflected in terms of the average gene expression that can be estimated (Shea and Ackers, 1985; Bintu et al., 2005b). Many of those models were tested with the average gene expression, which poorly represents the dynamic landscape in living systems (Shea and Ackers, 1985; Bintu et al., 2005a). This is because mean gene expression is independent of transcriptional dynamics. To elucidate the dynamics of transcriptional regulation, it is imperative to model different thermodynamic parameters when considering different stochastic transitions between credible promoter states (Austin et al., 2006; Hermsen et al., 2006; Sanchez et al., 2013; Yan et al., 2017). More inclusion of stochastic transitions across the time series must offer a better-informed description of the dynamics (Yan et al., 2017).

Robust mathematical modeling with fine resolution of quantitative factors, the inclusion of different parameters into a single framework, and the quality datasets altogether are essential standards to capture wide-spectrum dynamic snapshots of transcriptional events. Employing them may facilitate the reconstruction of accurate quantitative predictive models entailing strong stochastic insights about combinatorial transcriptional regulation.

### Stochasticity, Gene-Expression Noise, and Logic Gates

Stochastic variations in gene expression are primarily produced by a series of random transitions among different promoter states (Roberfroid et al., 2016). They are abundant in both prokaryotic and eukaryotic clonal populations and are used to generate random transcriptional variations, irrespective of external signals (Roberfroid et al., 2016). Two major types of noise have been identified: intrinsic and extrinsic noise (Figure 1B).

One possible origin of intrinsic noise is molecules that are dispersed and randomly collide within a cell (Golding et al., 2005; Baptista and Ribeiro, 2020). This type of noise arises from a short-duration event, such as the dissociation of a repressor from a promoter region that rebinds faster than an RNAP (van Zon et al., 2006; Walker et al., 2016). In such rapid rebinding, the rate of repressor dissociation diminishes and generates a degree of variation. The resultant variations in the rate of dissociation events result in noisy gene expression (van Zon et al., 2006; Walker et al., 2016). Therefore, the transcription rate influences the associated stochastic behavior. Genes with a lower transcription rate exhibited a higher occurrence of intrinsic noise. By accelerating the transcriptional rate, the intrinsic noise of a specific gene may get reduced and vice versa. However, extrinsic noise behaves differently and used to occur at intermediate transcription rates (Elowitz et al., 2002; Raj and van Oudenaarden, 2008). The interplay between different regulators is vital for noisy gene expression. How the regulators collaborate is crucial in determining the fate of gene expression within a cell (Choudhary and Narang, 2019; Torkaman and Jafarpour, 2019).

The mode of collaboration can be defined using the logic gate concept (Blake et al., 2006; Hermsen et al., 2006; Silva-Rocha and de Lorenzo, 2008; Warmflash and Dinner, 2008; Corrigan et al., 2016; Yan et al., 2017; Zoller et al., 2018; Bordoy et al., 2019; Iida et al., 2019). Logic gates rationalize different possible combinations of multiple regulators, including TFs and CREs, with their manifestations in gene expression (Lin et al., 2015; Yan et al., 2017; Larsson et al., 2019; Macauley et al., 2019). This is pivotal in explaining the generation of transcriptional noise in cells as they often tend to reduce the noise (Raj and van Oudenaarden, 2008). On the other side, noisy gene expressions are intermittently a positive phenomenon as it allows the cells to adapt extreme environments (Choudhary and Narang, 2019; Sarkisov et al., 2020).

### Transcriptional Burst Size, Frequency, and Exaggeration of Gene-Expression Noise

Cellular fitness and extreme adaptability used to rely on transcriptional noise, which is linked to transcriptional bursts. Transcriptional bursts include a range of molecular activities that occur during transcription and consider the number of states and activities at which a gene is transcribed (Zoller et al., 2018; Tunnacliffe and Chubb, 2020). Several models and hypotheses describe the characteristics of these bursts. The simplest is the “one-state” model that considers the transcriptional initiation rate as fixed and can influence the generation of fluctuations in transcriptional activity (Corrigan et al., 2016; Tunnacliffe and Chubb, 2020). However, this is not adequate for describing transcriptional dynamics. A two-state random telegraph model was subsequently proposed and has been widely adopted (Peccoud and Ycart, 1995). This model distinguishes the “active” and “inactive” modes of a transcriptional process, wherein a gene is typically transcribed once it is in an active state (Figures 2A,B). Fluctuations among these states are reproduced through mRNA synthesis in a bursty manner (Peccoud and Ycart, 1995). A small surge in mRNA synthesis may be interspersed with the period of inactivity (Raj et al., 2006; Tunnacliffe and Chubb, 2020). This bursting model is efficient for inferring transcriptional dynamics. It is also suitable in a genome-wide context, indicating global gene expression heterogeneity (Antolović et al., 2017) and provides mechanistic insights into transcriptional regulation (Larsson et al., 2019).

In bacteria, transcriptional bursts are quantified with a reporter gene, driven by the P*-lac/ara* promoter (Startceva et al., 2019), wherein the bursting duration follows a geometric distribution, separated by inactivation following an exponential distribution (Figures 2A,B). The on/off states of transcription can be modeled as a two-state random telegraph. The burst duration increases with an increase in the expression level, but the burst frequency and initiation rate remain constant (So et al., 2011; Startceva et al., 2019). The bacterial transcription can also be gene-independent (Lin et al., 2015; Baudrimont et al., 2017). The binding of an RNAP can be conceptualized as a series of on/off signals during the transcription process (Figures 2Aii), in which the “on” status corresponds to transcription initiation (Yan et al., 2017; Qiu et al., 2019). Therefore, the frequency of “on” events is equivalent to the rate of transcription initiation and the rate of mRNA synthesis, assuming every synthesized pre-RNA is converted into a mature mRNA (Ben-Tabou de-Leon and Davidson, 2009; Asif and Sanguinetti, 2013). Likewise, the TF-binding status can also be viewed as a digital signal with a series of “bind” and “unbind” events (Figures 2Aiii,v). The TF’s “bind” state lasts from the moment of TF binding to the time of TF-DNA dissociation. The duration of TF binding depends on the relative affinity of its competitors, such as DNA-binding molecules (Suter et al., 2011; Yan et al., 2017). However, the “unbound” state refers to the status wherein the promoter is not bound by the TFs. The “unbind” duration is the time that a TF spends searching for TFBS after TF-DNA dissociation. The mRNA synthesis rate is determined by the ratio of bursts to inactivation (Yan et al., 2017). Therefore, variations in bursting patterns may lead to variations in the levels of gene expression. The burst size is determined by the duration of TF binding, which is related to the affinity of TFs to their binding sites. The inactivation period between bursts is equal to the time that the TFs spend searching for their targets. Both burst size and inactivation periods are subjected to stochastic fluctuations due to the Brownian motion of molecules (Yan et al., 2017), which ultimately exaggerates gene-expression noise (Bokes and Singh, 2017).

## Discussion

Gene-expression noise is typically described as a driving force underpinning many cellular functions, rather than a kind of dysregulation of the central dogma. Single-cell and single-molecule studies have suggested that the fluctuations in transcriptional regulation may be governed under the direct control of cells in response to intracellular and extracellular signals. Direct observations with real-time transcription in living cells provide better spatial and temporal resolution, but they are limited in decoding the mechanisms of controlling noise and phenotypic heterogeneity (Satija et al., 2015; Goñi-Moreno et al., 2017; Zoller et al., 2018). A cell is capable of controlling diverse transcriptional patterns through combinatorial regulation by modulating the relative timing of two/multiple TFs upon constructing dynamic logic gates to regulate the target genes. However, a mechanistic explanation of how cells control transcriptional noise through combinatorial gene regulation is also not revealed. In this review, we have offered a snapshot of the latest developments, considerations, and perceptions in this area of synthetic biology.

Genetically identical bacterial cells behave differently despite growing together under identical conditions. Combinatorial gene regulation mediated by multiple TFs is extensively utilized in bacterial cellular programs in response to environmental conditions. A limited number of TFs are used to produce various expression patterns upon combinatorial interactions that diversely drives the gene transcription in bacteria. It is inherently a random process because of the stochastic behavior of TF molecules. These random fluctuations can potentially affect the functions of genetic circuits and downstream signaling pathways that lead to transcriptional noise in a population of cells. So, noise is a genome-wide phenomenon that is usually driven by the stochasticity of biochemical reactions occurring during gene expression. The synthesis of mRNA and proteins is used to follow an episodic bursting pattern during transcription and translation. Despite many studies, unraveling the mechanisms of transcriptional and translational noise generation, their associated consequences, and phenotypic variations remains challenging. Experiments guide us to decode a few instances of a phenomenon wherein the evolution in living systems is a larger combination of possibilities that are typically excluded with experimental set-up (Mejía-Almonte et al., 2020).

This work is concerned with one of the fundamental biological problems, noise in gene expressions in understanding the role of genetic circuits in cellular complexity and functions (Figure 1A). We emphasized the use of synthetic biocomputational approaches to create a holistic roadmap to understand not only the transcriptional noise-driven phenotypic variability but also its underlying regulatory logic. This may offer a path to purpose-driven programming of combinatorial regulation to augment phenotypic variability and adapt to fluctuating environments. Addressing them accurately is a challenge because of underdefined bacterial phenotypic heterogeneity. Genetically identical bacteria can behave differently despite exhibiting the same growth patterns due to variable gene expression patterns, which are noisy. The generation of such spontaneous dynamic performance is pervasive and has been used in microbes and mammalian cells. Hence, we evaluated the contradictory perceptions of the origin of such heterogeneity. However, figuring out the characteristics of variable noise is essential for genomics applications. Amidst the rising conflict between gene-specific and genome-wide transcriptional regulation, it is time to reconsider the scope, progression, and logical construction of the different viewpoints underlying the generation of gene-expression noise to reach a consensus on our scientific goal of understanding bacterial phenotypic heterogeneity. It is a consequence of randomness in gene circuits, which is tightly dictated by the transcription noise produced via combinatorial gene regulation. Static biochemical experiments are unable to explore the versatility and stochasticity of the transcriptional machinery. The shortfall between cutting-edge biophysics and current challenges must encourage researchers to develop better strategies to associate noise and related manifestations at the structural and functional levels, and eventually to reprogram combinatorial regulation to reverse-engineer the phenotypic variability against diverse adaptability.
